# Mitochondrial Complex I Inhibitors and Forced Oxidative Phosphorylation Synergize in Inducing Cancer Cell Death

**DOI:** 10.1155/2013/243876

**Published:** 2013-04-09

**Authors:** Roberta Palorini, Tiziana Simonetto, Claudia Cirulli, Ferdinando Chiaradonna

**Affiliations:** ^1^Department of Biotechnology and Biosciences, University of Milano-Bicocca, Piazza della Scienza 2, 20126 Milan, Italy; ^2^SysBio Centre of Systems Biology, Piazza della Scienza 2, 20126 Milan, Italy

## Abstract

Cancer cells generally rely mostly on glycolysis rather than oxidative phosphorylation (OXPHOS) for ATP production. In fact, they are particularly sensitive to glycolysis inhibition and glucose depletion. On the other hand mitochondrial dysfunctions, involved in the onset of the Warburg effect, are sometimes also associated with the resistance to apoptosis that characterizes cancer cells. Therefore, combined treatments targeting both glycolysis and mitochondria function, exploiting peculiar tumor features, might be lethal for cancer cells. In this study, we show that glucose deprivation and mitochondrial Complex I inhibitors synergize in inducing cancer cell death. In particular, our results reveal that low doses of Complex I inhibitors, ineffective on immortalized cells and in high glucose growth, become specifically cytotoxic on cancer cells deprived of glucose. Importantly, the cytotoxic effect of the inhibitors on cancer cells is strongly enhanced by forskolin, a PKA pathway activator, that we have previously shown to stimulate OXPHOS. Taken together, we demonstrate that induction in cancer cells of a switch from a glycolytic to a more respirative metabolism, obtained by glucose depletion or mitochondrial activity stimulation, strongly increases their sensitivity to low doses of mitochondrial Complex I inhibitors. Our findings might be a valuable approach to eradicate cancer cells.

## 1. Introduction

As indicated by Otto Warburg many years ago and now accepted as a hallmark of cellular transformation, cancer cells entirely reprogram their metabolism to sustain hyperproliferation and growth also in particular environmental conditions [1]. In particular, differently from normal cells, cancer cells rely mostly on glycolysis rather than oxidative phosphorylation (OXPHOS) for ATP production [2, 3]. Tumor environment, oncogenes, and tumor suppressor mutations have an important role in this energetic shift to aerobic glycolysis [4, 5]. Another important feature of metabolic reprogramming of transformed cells is their reduced or strongly impaired mitochondrial function [3, 6]. Despite that, mitochondria cover an important role also in cancer cells, that is, through the maintenance of mitochondrial potential and oxidative equilibrium, necessary for cell viability and apoptosis control, and for the different anabolic processes that use precursors produced in this organelle such as lipid, amino acids, and nucleotides synthesis. Thus, different therapeutic approaches have been addressed to cancer cell mitochondria. There is a series of compounds targeting mitochondria, named mitocans, that are being tested as anticancer drugs. They usually lead to cancer cell death by inducing mitochondria destabilization with a consequent increase of reactive oxigen species (ROS) and activation of apoptotic signals [7, 8]. Different classes of mitocans exist and can be classified into eight groups, more specifically hexokinase inhibitors, Bcl-2 homology-3 (BH3) mimetics, thiol redox inhibitors, drugs targeting the voltage-dependent anionic channel (VDAC) or the adenine nucleotide translocator (ANT), agents interfering with the electron transport chain (ETC), lipophilic cations targeting the inner membrane, agents interfering with the mitochondrial DNA, and drugs acting on not well-defined sites [8]. Among the compounds acting on the ETC, vitamin E analogues that in particular target Complex II have been tested as anticancer agents [9]. Complex I inhibitors have shown anticancer properties as well, for example the acetogenins, such as rollinistatin and bullatacin, and also rotenone itself, which exhibits antitumor activity in animal models [10]. 

On the other hand, cancer cells for their peculiar metabolism are particularly sensitive to treatments inhibiting glycolysis and to glucose deprivation [11, 12], since in both circumstances they lose hyperproliferative ability and ultimately die [12–15]. Therefore, combined treatment targeting both glycolysis and mitochondria, exploiting peculiar tumor features, may be lethal for cancer cells. In this regard it has been shown that cancer cells, like osteosarcoma cells, treated with ETC inhibitors, are induced to switch over to glycolysis becoming hypersensitive to the glycolytic inhibitors [16]. Equally, it has been shown that inhibition of glucose metabolism, for example, by using 2-deoxyglucose (2-DG), can make tumor cells more dependent on OXPHOS and therefore more sensitive to treatment with ETC inhibitors [17]. However, glycolytic inhibitors, like 2-DG, could be potentially toxic for tissues like the brain, retinae, and testis that use glucose as the main energy source. In addition, they are also not very potent and must be used at high concentrations [11]. 

In a previous study it has been shown that treatment of cancer cells with dichloroacetate (DCA), a TCA cycle inducer, is able to redirect their metabolism from glycolysis to oxidative phosphorylation and hence to lead them towards apoptosis [18]. Therefore, it has been supposed that induction of a reversion of the Warburg effect coupled to a treatment able to interfere with mitochondrial activity could specifically kill cancer cells. Recently we have shown that exogenous activation of PKA pathway can improve several mitochondrial parameters, leading to a Warburg effect reversion, in K-ras cancer cells, where the Protein Kinase A (PKA) pathway is generally deregulated [19]. In fact, cancer cells treated with forskolin (FSK), an activator of adenylate cyclase [20], show an increase of Complex I activity, an increase of mitochondrial ATP production, a decrease of ROS generation, and an increase of mitochondria interconnections, that may lead to survival under glucose depletion [15].

Since nutrient deprivation widely exists in solid tumors because of the poor blood supply [21, 22], we decided to study the effects on cancer cells of glucose depletion, mimicking physiological tumor condition, instead of glycolysis inhibitors, combined with treatments with OXPHOS Complex I inhibitors. As results we demonstrate that in low glucose availability different cancer cell lines, in a way dependent on their glycolytic metabolism, become sensitive to short treatment with low doses of Complex I inhibitors as compared to optimal glucose condition. In fact we observe an increased cell death. Interestingly, in such a glucose-depleted condition, we also find evidence that stimulation of mitochondrial activity by FSK can further sensitize cancer cells to Complex I inhibitors by enhancing cancer cell death. Altogether our findings indicate that stimulation of respiratory chain activity, in low glucose availability, makes glycolytic cancer cells more sensitive to OXPHOS inhibitors.

## 2. Material and Methods

### 2.1. Cell Cultures

Breast cancer cells MDA-MB-231, mouse fibroblasts NIH3T3 Normal and Transformed, pancreatic cancer cells MIA PaCa-2, and lung cancer cells A549 [15] were routinely cultured in Dulbecco's modified Eagle's medium (DMEM) containing 4 mM L-glutamine, 100 U/mL penicillin, and 100 mg/mL streptomycin (complete medium), supplemented with 5–10% fetal bovine serum (human cells) or 10% newborn calf serum (mouse cells). For the experiments cells were plated in complete growth medium. After 16 hours cells were washed twice with phosphate buffer saline (PBS) and incubated in growth medium (time 0) without glucose and sodium pyruvate, supplemented with 25 or 1 mM glucose. Treatments and analyses were performed at 48 hours (MDA-MB-231 and A549) or 72 hours (NIH3T3 and MIA PaCa-2) after time 0. All reagents for media were purchased from Life Technologies (Carlsbad, CA, USA).

### 2.2. Treatments

Rotenone, oligomycin, and FSK were purchased from Sigma-Aldrich Inc. (St. Louis, MO, USA). Capsaicin and piericidin A were purchased from Vinci-Biochem (Florence, Italy).

### 2.3. Viability Assays

Cell viable count was performed by staining cells with Trypan Blue 0.4% (Life Technologies).

Propidium iodide (PI)/Annexin V-FITC staining was performed using Apoptosis Assay Kit from Immunological Sciences (Rome, Italy) and analyzed by FACScan flow cytometer (Becton-Dickinson, Franklin Lakes, NJ, USA) with CellQuest software (Becton-Dickinson). Flow cytometric data were then carried out using the freely available WinMDI software.

For the evaluation of PI incorporation 5 × 10^5^ cells were harvested and stained with 5 *μ*g/mL PI and 5 *μ*g/mL Hoechst (Sigma-Aldrich Inc.) in PBS for 15 min at r.t. After staining, cells were mounted on a microscope slide with 50% glycerol and analyzed under a Nikon ECLIPSE 90i fluorescence microscope (Nikon, Tokyo, Japan) equipped with a b/w CCD camera (Hamamatsu-CoolSNAP, Hamamatsu Corporation, Hamamatsu City, Japan). The images were acquired using the imaging software Metamorph 7 and then visualized and processed in Image J (freely available).

### 2.4. Clonogenic Assay

For each sample 3 × 10^3^ cells were plated in 100 mm dish. After ≥12 days colonies were fixed with PBS-formaldehyde 5%, stained with crystal violet 1%, and then counted.

### 2.5. Intracellular ATP and Mitochondrial Potential Quantification

Intracellular ATP levels were measured using CellTiter Glo luciferin-luciferase assay (Promega, Madison, WI, USA) as described in [23].

Mitochondrial potential was analyzed by staining cells with 20 nM JC-1 (5,5′,6,6′-tetrachloro-1,1′,3,3′-tetraethylbenzimidazolylcarbocyanine iodide, Life Technologies) for 10 minutes. After staining, flow cytometric analysis was performed acquiring FL1 (JC-1 monomers, low potential) and FL2 (JC-1 aggregates, high potential) signals. For each sample the ratio FL2/FL1 was calculated and used to compare different samples.

### 2.6. D-Glucose Measurement

D-Glucose levels in culture medium were determined using a spectrophotometric assay kit (R-Biopharm, Darmstadt, Germany) as specified by manufacturer's datasheet.

### 2.7. Western Blot Analysis

For the analysis of cleaved Caspase 3 and Actin B expression, cells were harvested and lysed in Laemli buffer (50 mM Tris-HCl pH6.8, glycerol 6%, SDS 2%, *β*-mecaptoethanol 5%, bromophenol blue 0.05%). Samples were then resolved by sodium dodecyl sulfate polyacrylamide gel electrophoresis and transferred to nitrocellulose membrane, which was incubated overnight with antibodies for cleaved Caspase 3 (Cell Signaling Technology Inc., Danvers, MA, USA; 1 : 1000) and Actin B (Abcam, Cambridge, UK; 1 : 1000).

### 2.8. Oxygen Consumption Rate (OCR) Measurement

Oxygen consumption was determined using Seahorse XF24 extracellular Flux analyzer (Seahorse Bioscience, North Billerica, MA, USA). Cells were seeded in the 24-well XF24 cell culture plate in the culture medium containing 25 or 1 mM glucose, as described above. Where indicated, cells were also treated with FSK. Culture media were exchanged for base media (unbuffered DMEM supplemented with 10 mM sodium pyruvate and 20 mM glucose for cells grown in high glucose or only 10 mM sodium pyruvate for cells grown in low glucose) 1 hour before the assay and for the duration of the experiment. Selective inhibitors were injected during the measurements to achieve final concentrations of rotenone 3 nM and piericidin A 5 nM. The baseline OCR was defined as the average of the values measured from time points 1 to 5 (0–45 min) during the experiments. Due to some variations in the absolute magnitude of OCR measurements in different experiments, the relative OCR levels were used to compare and summarize independent biological replicates. After the analysis the cells were fixed, stained with Crystal Violet, and dosed at spectrophotometer after colorant solubilization with acetic acid 10%; all OCR values obtained by the instrument were normalized on cell density.

## 3. Results

### 3.1. Complex I Inhibition by Rotenone Influences Cancer Cell Survival Depending on Initial Glucose Availability

**Figure 1:**
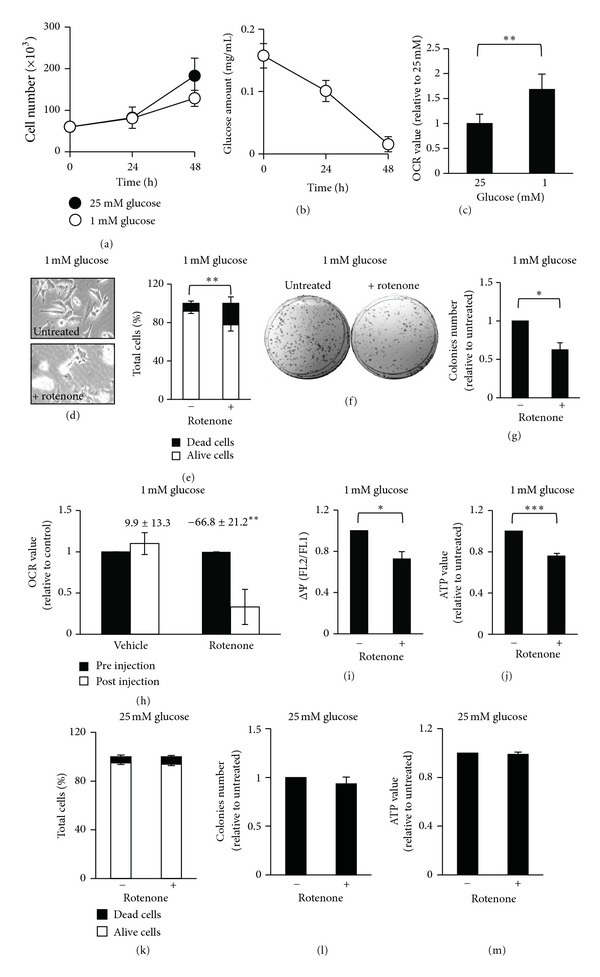
MDA-MB-231 cells are sensitive to rotenone in condition of glucose deprivation. (a) Proliferation curves for MDA-MB-231 cells cultured at 25 and 1 mM glucose were obtained counting cells at indicated time points. (b) Glucose amount in medium of cells cultured in 1 mM glucose was measured using enzymatic kit at indicated time points. (c) Basal OCR of MDA-MB-231 cells grown in 25 and 1 mM glucose was determined by Seahorse XF24 analyzer; data represent the average ± s.e.m. of three independent experiments (total number of samples ≥ 10), **P < 0.01 (Student's *t*-test). (d)–(j) MDA-MB-231 cells cultured for 48 hours in 1 mM glucose were treated for 4 hours with 3 nM rotenone. After treatment, optical microscopy images (d) and viable cell count performed by using Trypan Blue Staining (e) were obtained for untreated (−) and treated (+) cells. After treatment 3 × 10^3^ cells were also plated in normal growth medium for clonogenic assay and after ≥12 days colonies were stained (f) and counted (g). OCR of MDA-MB-231 cells cultured in low glucose was determined by Seahorse XF24 analyzer 30 minutes after injection of vehicle or 3 nM rotenone; the percentage of OCR variation after injection is reported (h). In untreated (−) and treated (+) cells also mitochondrial potential (ΔΨ) indicated as ratio of mean fluorescence FL2 on mean fluorescence FL1 (see Section 2) (i) and intracellular ATP levels (j) were measured. (k)–(m) Cell count (k), clonogenic assay (l), and intracellular ATP measurement (m) were performed also in cells grown for 48 hours in 25 mM glucose and treated with rotenone as above. All data represent the average of at least three independent experiments (±s.d.); *P < 0.05, **P < 0.01, ***P < 0.001 (Student's *t*-test).

MDA-MB-231 human breast cancer cells, like several other cancer cells, use mainly glycolysis instead of mitochondrial respiration to generate ATP and other anabolic substrates necessary for their proliferation and survival [24]. In fact, in low glucose availability, these cells show a reduced proliferation and an increase of cell death because of their inability to maximize the use of OXPHOS especially for energetic use [15]. In this scenario, we tested the ability of rotenone, an inhibitor of OXPHOS, to increase their sensitivity to glucose depletion. Rotenone is a natural compound that has been used to interfere with mitochondrial respiration, in particular with Complex I activity, and hence to reduce intracellular ATP levels especially in OXPHOS-dependent cell lines [25, 26]. In order to evaluate their ability to proliferate and survive under OXPHOS inhibition, we treated proliferating MDA-MB-231 cells, grown for 48 hours in low glucose (1 mM glucose, 4 mM glutamine) or high glucose (25 mM glucose, 4 mM glutamine), with rotenone. The treatment was executed at 48 hours of culture because, despite a comparable proliferation rate in the two different glucose concentrations (Figure 1(a)), in low glucose condition external medium analysis indicated that this carbon source was almost completely depleted at this time point (Figure 1(b)). In addition, measurement of the basal cellular oxygen consumption rate (OCR) by Seahorse XF analyzer indicated a 40% increase of cellular respiration rate in cells grown in low glucose (Figure 1(c)), suggesting that MDA-MB-213 cells, in absence of glucose as main substrate for glycolysis, partially shifted from glycolysis to mitochondrial respiration. Short treatment with a low concentration of rotenone (3 nM for 4 hours), known to be ineffective on normal cell mitochondria activity [27–29], in glucose-depleted condition induced a reduction of cell viability, as confirmed by morphological analysis (Figure 1(d), circle and floating cells) and by Trypan Blue viable cell count (Figure 1(e)). In fact, after treatment Trypan Blue-positive cells increased from 10.2% to 22.3%. This effect on cell survival was also supported by clonogenic assays (Figures 1(f) and 1(g)), which showed that treated cells, replated in high glucose condition (25 mM), formed less colonies (about 50% of reduction) as compared to untreated control. Such an assay shows that the short treatment is enough to reduce cancer cell ability to form a large colony and proliferate, suggesting that rotenone, inhibiting the alternative mitochondrial energetic route of these cancer cells upon glucose deprivation, heavily affects their viability. Rotenone outcome on mitochondrial activity was evaluated by determination of OCR, mainly due to mitochondrial respiration, mitochondrial potential, and intracellular ATP levels. In particular, 3 nM rotenone was injected by the instrument into the cells and its effect analyzed between 30 minutes and 1 hour after the injection. As shown in Figure 1(h), OCR was reduced to ~30% of the baseline rates, indicating that 3 nM rotenone is able to decrease mitochondrial respiration. Also mitochondrial potential was reduced by rotenone (Figure 1(i)), confirming the direct effect of the treatment on mitochondrial function. In the same experimental setting, rotenone induced also a ~25% decrease of the intracellular ATP levels (Figure 1(j)).

Importantly, rotenone treatment in nonlimiting glucose condition had no effect on cell survival, as confirmed by Trypan Blue viable cell count (Figure 1(k)) and clonogenic assay (Figure 1(l)). Moreover, rotenone had no effect on intracellular ATP levels (Figure 1(m)), suggesting that in high glucose availability ATP is generated essentially by glycolysis.

### 3.2. Under Glucose Depletion, Rotenone and FSK Cause Enhanced Mouse K-Ras-Transformed Cell Death as Compared to Immortalized Counterpart

 It has been proposed that Warburg effect reversion, gained by mitochondrial reactivation, may be a promising method for promoting naturally encoded programmed cell death and hence kill cancer cells [18]. Given that, we sought to investigate whether the combination of FSK, able to restore mitochondrial activity, and rotenone could synergistically enhance the killing of cancer cells in glucose depletion. First, we performed such an analysis on NIH3T3 mouse fibroblasts (immortalized cells, Normal), an OXPHOS-dependent cell line, and NIH3T3 mouse fibroblasts expressing an oncogenic *K-RAS* gene (Transformed) [15, 24].

The latter cellular model of transformation is suitable since it presents a transcriptional profile and different metabolic features, such as the Warburg effect, comparable to several human cancer cells harboring an oncogenic *K-RAS* gene, like, for instance, MDA-MB-231 cells [23, 24, 30, 31]. 

**Figure 2:**
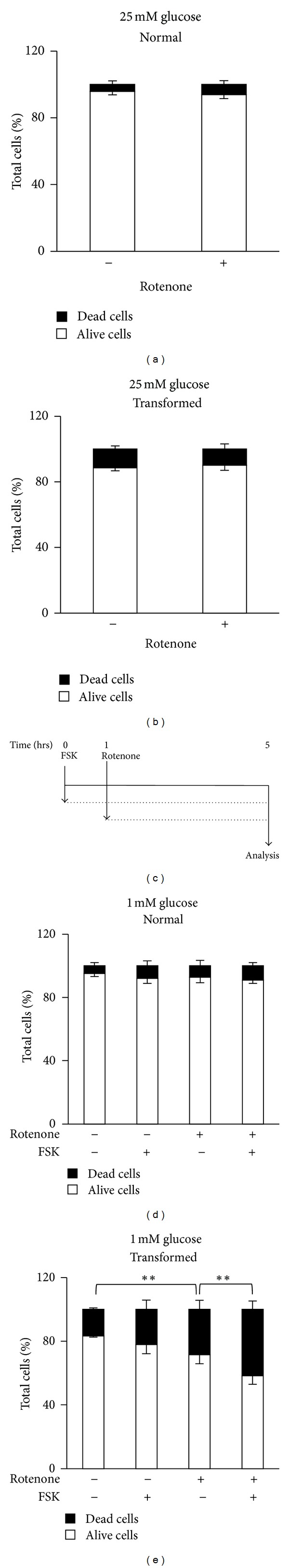
Low doses of rotenone do not affect Normal cells survival as compared to Transformed cells. Viable cell count using Trypan Blue was performed after treatment with rotenone at 72 hours of culture in different growth conditions. (a)-(b) NIH3T3 (a) Normal and (b) Transformed cells were cultured in 25 mM glucose and counted after 4-hour treatment with 3 nM rotenone. (c)–(e) Cells were cultured in 1 mM glucose and treated with 3 nM rotenone, 10 *μ*M FSK, or both molecules. For the combined treatment, cells were pretreated for 1 hour with FSK and then rotenone was also added for 4 hours as represented in (c). After treatment (d) Normal and (e) Transformed cells were counted. All data represent the average of at least three independent experiments (±s.d.); **P < 0.01 (Student's *t*-test).

The cells grown for 72 hours in both initial glucose concentrations were incubated with rotenone and FSK alone or in combination. As shown in Figures 2(a) and 2(b), in nonlimiting glucose condition rotenone had no effect on proliferation of both cell lines, confirming that such a low rotenone concentration does not inhibit mitochondrial respiration of Normal cells (Figure 2(a)) and does not induce cell death in mouse Transformed cells (Figure 2(b)), as previously observed in MDA-MB-231 cells. On the contrary, cells grown in low glucose for 72 hours, the time point at which both cell lines have completely consumed the glucose in the culture medium [15], showed a different response to the treatments with rotenone and/or FSK (refer to Figure 2(c) for treatments schedule). Normal cells were found to be insensitive to rotenone either alone or in combination with FSK (Figure 2(d)). In contrast, Transformed cells showed 17% of cell death in basal condition, 22% upon FSK treatment, 29% upon rotenone, and 42% when the two compounds were used in combination (Figure 2(e)). These data indicate that Normal mouse cells, relying especially on mitochondrial respiration, are less responsive to low doses of rotenone as well as to the combined treatment with FSK. On the contrary, Transformed mouse cells, forced to use mitochondrial respiration by glucose deprivation or FSK treatment, become more sensitive to the Complex I inhibitor.

### 3.3. Under Glucose Depletion the Combined Treatment with Rotenone and FSK Leads to an Increase of MDA-MB-231 Cell Death as Compared to Rotenone Alone

**Figure 3:**
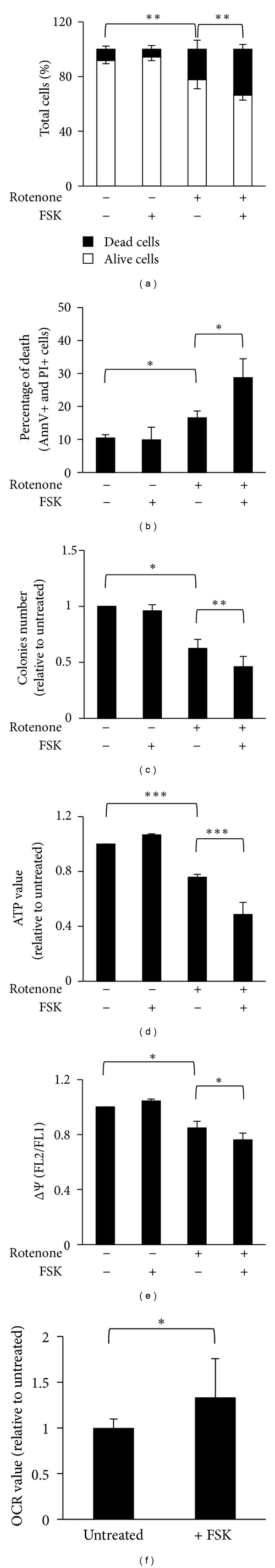
FSK treatment enhances the viability loss induced by rotenone alone in MDA-MB-231 cells. MDA-MB-231 cells were cultured in 1 mM glucose and treated with 3 nM rotenone, 10 *μ*M FSK, or both molecules at 48 hours of culture. Cells were pretreated for 1 hour with FSK and then rotenone was also added for 4 hours, as shown in Figure 2(c). After treatment different parameters were investigated in untreated (−) and treated (+) cells. Viable cell count was performed using Trypan Blue (a). Percentage of cell death was evaluated after staining with PI and Annexin V-FITC (AnnV). Cells positive for one or both molecules were considered dead cells (b). After treatment 3 × 10^3^ cells were plated in normal growth medium for clonogenic assay and after ≥12 days colonies were stained and counted as reported in the histogram (c). (d) Intracellular ATP levels and (e) mitochondrial potential were also measured. All data represent the average of at least three independent experiments (±s.d.); *P < 0.05, **P < 0.01, ***P < 0.001 (Student's  *t*-test). (f) Basal OCR of MDA-MB-231 cells, grown 1 mM glucose and treated or not with FSK for 2–4 hours, was determined by Seahorse XF24 analyzer. Analysis was performed in three independent experiments (total number of samples ≥ 15) and values are indicated as the average ± s.e.m.; *P < 0.05 (Student's  *t*-test).

Since a previous study has indicated that MDA-MB-231 cells are responsive to FSK treatment as well as mouse K-ras-transformed fibroblasts [15], we treated MDA-MB-231 cancer cells, grown in low glucose, with rotenone and FSK alone or in combination (schedule is shown in Figure 2(c)). In order to evaluate cell death upon single or combined treatments, the cells were analyzed through Trypan Blue viable count (Figure 3(a)) or PI/Annexin V staining followed by FACS analysis (Figure 3(b) and Supplementary Figure 1 in the Supplementary Material available online at http://dx.doi.org/10.1155/2013/243876). As shown in Figures 3(a) and 3(b), both analyses indicated an increased cell death in the samples subjected to the combined treatment as compared to either untreated or rotenone-alone-treated samples. In particular, Trypan Blue Staining indicated an increase of positive cells from 22% for rotenone alone to 33.6% in presence of FSK. Similar values were observed by PI/Annexin V staining (18% rotenone versus 30% rotenone + FSK). Importantly, the combined treatment further reduced cancer cell ability to form colonies as compared to rotenone alone (Figure 3(c)). The increase of cell death was associated with a reduction of about 50% of intracellular ATP levels (Figure 3(d)) and of around 20% of mitochondrial potential (Figure 3(e)) as compared to untreated samples. To confirm a role of FSK in inducing a positive effect on mitochondrial activity, next we measured basal OCR, as previously described, in untreated or 2-to-4-hour FSK-treated cells. The interval of treatment was chosen since in our assays the cells were treated with FSK (pretreatment plus combination with OXPHOS inhibitors) for a maximum time of 5 hours. As shown in Figure 3(f), FSK-treated samples showed an increase of around 30% of OCR as compared to untreated samples, suggesting that the formers are more respirative than the latter ones. Moreover we did not observe differences in OCR values obtained in 2 and 4 hours-treated samples. Altogether these findings indicate that, upon glucose depletion, the stimulation of respiratory chain activity makes cells more sensitive to OXPHOS inhibitors.

### 3.4. Piericidin A and Capsaicin, Inhibitors of Mitochondrial Complex I, Show the Same Effects of Rotenone and Synergize with FSK in Inducing MDA-MB-231 Cell Death

**Figure 4:**
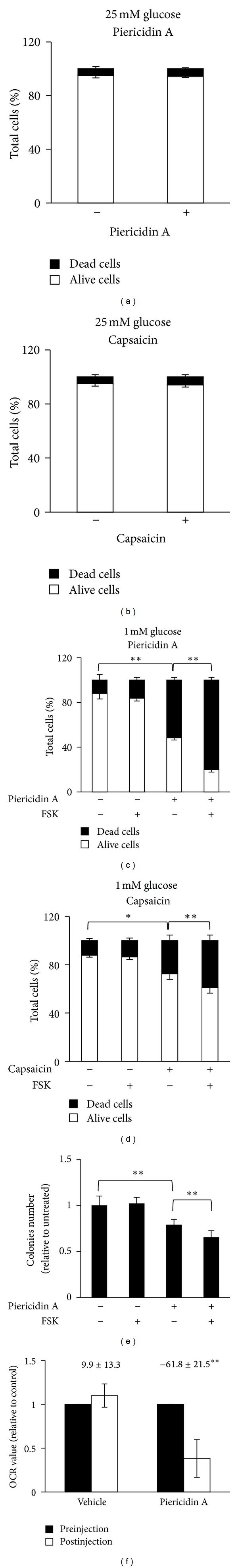
The mitochondrial Complex I inhibitors piericidin A and capsaicin induce cell death in MDA-MB-231 cells as observed with rotenone. Viable cell count using Trypan Blue was performed after treatment with 5 nM piericidin A or 100 *μ*M capsaicin at 48 hours of culture in different growth conditions. (a)-(b) MDA-MB-231 cells were cultured in 25 mM glucose and counted after 2-hour treatment with piericidin A (a) or capsaicin (b). (c)-(d) MDA-MB-231 cells were cultured in 1 mM glucose and treated with (c) piericidin A or (d) capsaicin, 10 *μ*M FSK, or FSK together with Complex I inhibitors. In the last case, cells were pretreated with FSK for 1 hour and then piericidin A or capsaicin were added for 2 hours (see Figure 2(c) as example). After treatment cell count was performed. (e) After treatment with piericidin A 3 × 10^3^ cells were plated in normal growth medium for clonogenic assay and after ≥12 days colonies were stained and counted. (f) OCR of MDA-MB-231 cells cultured in low glucose was determined by Seahorse XF24 analyzer 30 minutes after injection of vehicle or 5 nM piericidin A; the percentage of OCR variation after injection is reported. All data represent the average of at least three independent experiments (±s.d.); *P < 0.05, **P < 0.01 (Student's  *t*-test).

To further confirm the role of mitochondrial inhibition in the cell death mechanism upon glucose depletion, and more specifically the role of Complex I, we used two other inhibitors of this complex, namely piericidin A and capsaicin [32]. Importantly piericidin A, differently from rotenone that at higher concentration may affect cell cycle [33, 34], does not interfere with the cell cycle execution. As shown in Figures 4(a) and 4(b), upon 2 hours of treatment, both inhibitors, as previously observed with rotenone, did not induce cell death when added to MDA-MB-231 grown in high glucose. On the contrary, their addition to glucose-depleted cells led to an increase of MDA-MB-231 cell death that was much stronger in the samples treated with piericidin A (52%) (Figure 4(c)) than with capsaicin (28%) (Figure 4(d)). Notably, combined treatment with FSK further increased the percentage of cell death that reached a value of 80% in the sample piericidin A + FSK (Figure 4(c)) and 39% in the sample capsaicin + FSK (Figure 4(d)).

Since cell viability was greatly affected by piericidin A, we evaluated also the potential of this molecule in combination with FSK in clonogenic assays (Figure 4(e)). Obtained data indicated a significant reduction of colonies number after treatment with piericidin A, reduction that was further increased upon combination with FSK as compared to untreated and FSK-treated sample (Figure 4(e)). Notably, OCR measurement indicated that piericidin A was able to decrease mitochondrial respiration (Figure 4(f)) as well as previously observed with rotenone (Figure 1(h)), confirming its effect on cellular mitochondrial respiration.

### 3.5. FSK Enhances Complex I Inhibitors Effect on Cancer Cell Viability by Increasing Necrotic Cell Death

**Figure 5:**
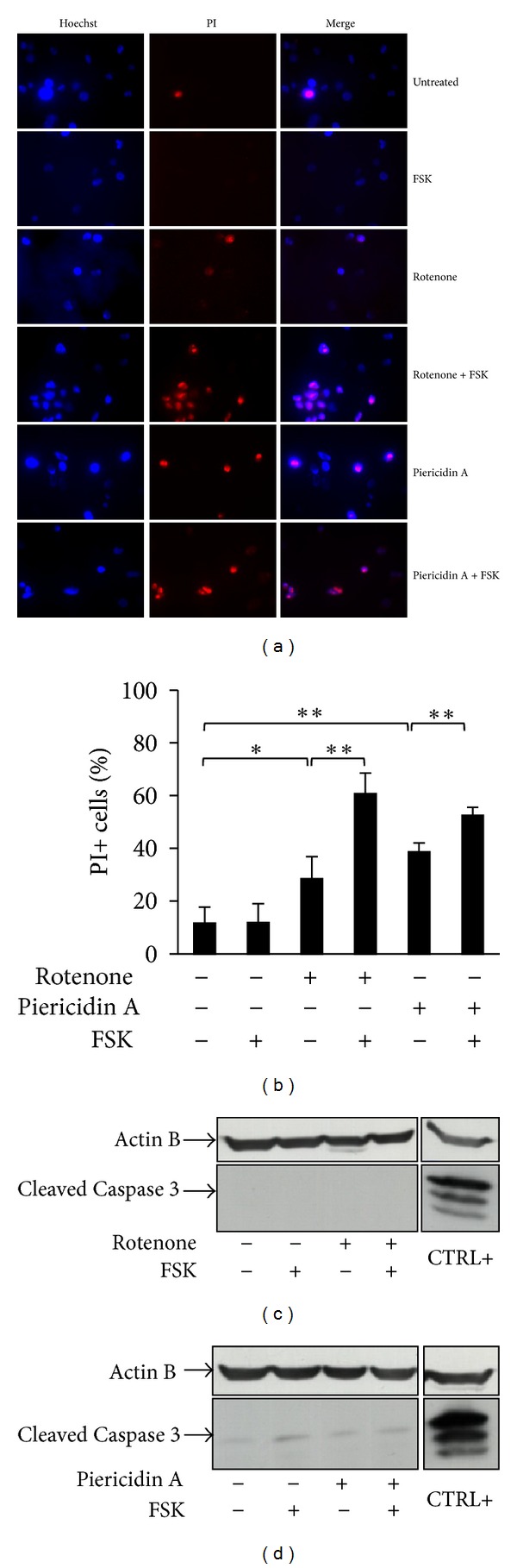
The combined treatment with Complex I inhibitors and FSK induces necrosis of MDA-MB-231 cells. Analysis of cell death was performed for MDA-MB-231 cells grown in low glucose and treated with 3 nM rotenone and 5 nM piericidin A alone or in combination with FSK. (a)-(b) Cells were stained with Hoechst and PI (a). As indication of necrosis cells that incorporated PI were counted and shown as percentage of the total cells (stained with Hoechst) considering at least 100 cells per sample (b). Data represent the average of at least three independent experiments (±s.d.); *P < 0.05, **P < 0.01 (Student's  *t*-test). (c)-(d) As indication of apoptosis the expression of the cleaved Caspase 3 was analyzed in cells treated with (c) rotenone and (d) piericidin A. As apoptotic control MDA-MB-231 cells were treated with thapsigargin (CTRL+). As loading control the expression of Actin B was evaluated. Data are representative of three independent experiments.

Glucose deprivation has been shown to kill cells either by necrosis or through the mitochondrial pathway of apoptosis [35]. Similarly, prolonged treatment with mitochondrial OXPHOS inhibitors also lead to necrotic cell death [36]. In order to assess whether single or combined treatments could induce necrosis or apoptosis, we performed experiments of PI incorporation followed by microscopy analysis and of Western blot. As shown in Figures 5(a) and 5(b), Complex I mitochondrial inhibitors (rotenone and piericidin A) caused an increase of PI incorporating cells as compared to untreated or FSK-treated samples. As expected from previous results, FSK treatment further enhanced the percentage of PI incorporating cells, supporting the notion that cells were experiencing a necrotic cell death process (Figures 5(a) and 5(b)). Such a cell death mechanism was confirmed by morphology analysis indicating cell detachment (data not shown) and plasma membrane damage without nuclear condensation (Figure 5(a), Hoechst staining). In addition, this cell death process was not associated with activation of Caspase 3 (Figures 5(c) and 5(d)) and poly(ADP-ribose) polymerase (PARP) (data not shown), both considered apoptotic markers, as it was seen with thapsigargin treatment for 6 hours (Figures 5(c) and 5(d)). 

### 3.6. Mitochondrial Complex I Inhibitor Piericidin A Synergize with FSK in Inducing Death of MIA PaCa-2 and A549 Cancer Cells

**Figure 6:**
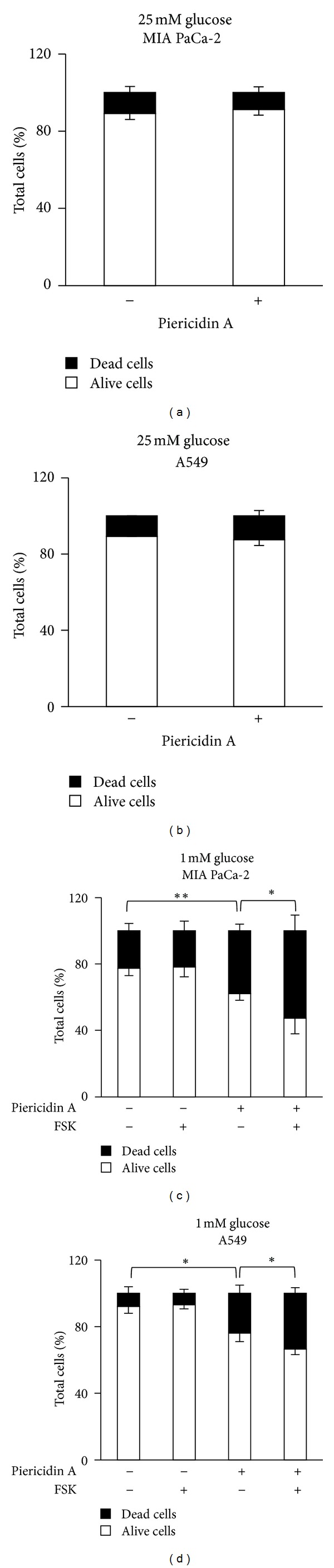
Piericidin A and low glucose synergize in inducing death also of pancreatic and lung cancer cells. Viable cell count using Trypan Blue was performed after treatment with 10 nM piericidin A in different growth conditions. (a)-(b) MIA PaCa-2 (pancreatic, A) and A549 (lung, B) cancer cells were cultured in 25 mM glucose and counted after 8-hour treatment. (c)-(d) MIA PaCa-2 (c) and A549 (d) cells were cultured in 1 mM glucose and treated with piericidin A, 10 *μ*M FSK, or FSK plus piericidin A. For the cotreatment experiments, cells were pretreated with FSK for 1 hour and then piericidin A was added for 8 hours (see Figure 2(c), as example). After treatment cell count was performed. All data represent the average of at least three independent experiments (±s.d.); *P < 0.05, **P < 0.01 (Student's  *t*-test).

To examine whether other cancer cell lines showed similar sensitivity to Complex I inhibitors alone and combined with FSK, we examined the effect of the treatments on two different human cancer cell lines with a different dependence on glucose availability. Previous data, in fact, have shown that MIA PaCa-2 (pancreatic cancer cell line) and A549 (non-small-cell lung cancer cell line) are both sensitive to glucose deprivation, in particular MIA PaCa-2, undergoing cell death, and, in different extend, are both protected by FSK treatment because of its ability to restore Complex I activity [15]. Moreover, both cell lines express an oncogenic K-ras that has been shown to promote cell metabolic rewiring and in particular glycolysis [24, 37]. As shown in Figures 6(a) and 6(b) Piericidin A treatment, used at concentration of 10 nM for 8 hours, did not affect survival of both cell lines grown in 25 mM glucose, as measured by Trypan Blue vital staining. On the contrary, as previously observed for MDA-MB-231 cells, its addition in glucose depletion led in both cell lines to an increase of cell death that was stronger in more glycolytic cells MIA PaCa-2 (37.9%) (Figure 6(c)) than in A549 (23.9%) (Figure 6(d)). Notably, combined treatment with FSK further increased the percentage of cell death, reaching a value of 52.6% in MIA PaCa-2 (Figure 6(c)) and 33.4% in A549 (Figure 4(d)). Interestingly, in MIA PaCa-2 cells an increase of cell death was already observed in cells grown in low glucose as compared to high glucose (22.6% versus 10.8%), confirming their major dependence on glucose availability as compared to A549 cells.

### 3.7. Oligomycin-Dependent Cell Death of MDA-MB-231 Cells Increases in Glucose Deprivation

**Figure 7:**
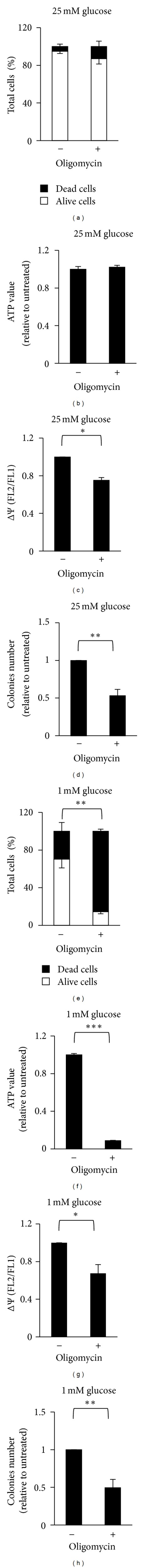
Oligomycin treatment induces a decrease of MDA-MB-231 cell viability especially in low glucose growth. MDA-MB-231 cells were cultured in (a)–(d) 25 mM and (e)–(h) 1 mM glucose and treated for 1 hour with 5 *μ*M oligomycin at 48 hours of culture. After treatment different parameters were investigated in untreated (−) and treated (+) cells: viable cell count by (a), (e) Trypan Blue Staining, (b), (f) intracellular ATP levels, and (c), (g) mitochondrial potential. After treatment 3 × 10^3^ cells were also plated in normal growth medium for clonogenic assay and after ≥12 days colonies were stained and counted (d), (h). All data represent the average of at least three independent experiments (±s.d.); *P < 0.05, **P < 0.01, ***P < 0.001 (Student's *t*-test).

To further confirm the major sensitivity of cancer cells to inhibitors of mitochondrial function in a condition of low glucose, we decided to use also the mitochondrial ATP synthase inhibitor oligomycin. MDA-MB-231 cells are considered a cell line with a high glycolytic rate and a low level of respiration [15, 24]. For this reason we supposed that under treatment with oligomycin these cells could undergo a limited effect in high glucose and a significant effect in low glucose, since the latter condition is characterized by acute stimulation of respiration. MDA-MB-231 cells, cultured in 25 mM glucose, were treated for 1 hour with 5 *μ*M oligomycin and then analyzed. As shown in Figures 7(a) and 7(b) both cell viability and total intracellular ATP were not changed by the treatment, whilst a slight decrease of mitochondrial potential and a consistent decrease of colony formation ability were observed (Figures 7(c) and 7(d)). On the other hand, MDA-MB-231 cells grown in low glucose and treated with oligomycin showed a strong increase of cell death as indicated by Trypan Blue viable count (Figure 7(e)). Moreover, a complete depletion of intracellular ATP (Figure 7(f)), associated with a partial reduction of mitochondrial potential (Figure 7(g)), was observed. All these parameters were accompanied by a further decrease in the ability to form colonies as compared to cells grown in high glucose (Figure 7(h)). Taken together, these data indicate that in glucose shortage glycolytic cancer cells show a stronger and forced dependence on mitochondrial respiration that make them very sensitive to inhibitors of mitochondria function.

## 4. Discussion

Recently, different therapeutic approaches based on targeting tumor mitochondria have been proposed [8]. In fact, since this organelle is central both as producer of cellular ATP and as central regulator of apoptosis, it may be considered a good therapeutic hit [38]. The primary metabolic function of mitochondria is OXPHOS, an energy-generating process that couples the oxidation of respiratory substrates with ATP production. Besides ATP synthesis, mitochondria are involved in several other key metabolic processes such as oxidative decarboxylation of pyruvate, tricarboxylic acid cycle, and fatty acids oxidation. In addition, mitochondria take part in intracellular homeostasis of calcium and phosphate as well as in the balance of NAD^+^/NADH. Besides their central role in metabolic activity, more recently mitochondria have been shown to have a central role also in the cascade of events that leads to programmed cell death. In fact mitochondria represent a central checkpoint of this process by integrating various signals coming from endogenous factors (ions, metabolites, second messengers), from endogenous signaling proteins (kinases and phosphatases), and from exogenous factors (nutrients, oxygen). Therefore the strong interconnection between metabolism and apoptosis and the central role of mitochondria in both processes have led to an explosion of interest in connecting such pathways to the pathophysiology of cancer. 

In this report, we have decided to utilize the main metabolic alterations of cancer cells, namely hyperglycolytic phenotype (Warburg effect) and mitochondria dysfunction, as targets for combined treatments aimed to specifically kill cancer cells. In fact, as shown by a number of groups, cancer cell proliferation and tumor aggressiveness correlate with an enhanced glycolysis and a low mitochondrial respiratory chain activity [3, 31], and positive or negative modulation of OXPHOS activity, depending on the metabolic state of cancer cells, appears to reduce tumor growth [39, 40]. Herein by using a glycolytic human breast cancer cell line, namely MDA-MB-231, grown in limiting glucose availability, and some natural inhibitors of mitochondria activity, we show that Complex I inhibition associated with an acute stimulation of respiration, due to glucose depletion, induces specifically necrotic cancer cell death (Figures 1, 3, and 5). Notably, this finding has been observed by using three different Complex I inhibitors [32] that strongly support our results (Figures 4 and 5). Moreover, we show that this cell death process, induced by the treatment with Complex I inhibitors, is activated also in other three cancer cell lines, mouse K-ras transformed fibroblasts, human MIA PaCa-2 pancreatic cancer cells, and human A549 lung adenocarcinoma cells. Importantly, such cell death mechanism is strongly dependent on glycolytic rate of the cancer cells. In fact MIA PaCa-2 cells, known to have a higher glycolysis as compared to A549, appear to be more sensitive to the treatment with inhibitors (Figure 6). Our results are interesting also because our and previously published data indicate that, at the used concentrations, rotenone (3 nM), capsaicin (100 *μ*M), and piericidin A (5 nM) have no effect on immortalized fibroblasts (Figure 2), on normal pancreatic cells [41] and on dopamine neurons [28], respectively. This reduced or absent effect on normal cells is an important characteristic for exploiting these compounds for cancer therapy. Regardless of the mechanism of action of the three molecules, we show that the sensitivity to them increases upon glucose depletion that reflects the dependency of the cells on glycolysis. We suppose that less glycolytic cells, such as immortalized fibroblasts or normal cells, will be also less sensitive to these treatments. Since we observed a necrotic cell death (Figure 5), we attribute the synergistic effects more to ATP depletion than a rapid decrease of mitochondrial potential. However we cannot exclude an increase of ROS levels, as shown by other authors, as a consequence of Complex I inhibition [17, 25]. In fact, it is possible that stimulation of mitochondrial activity upon glucose depletion in the deranged respiratory system of malignant cells results in an increased production of oxidants, which may overwhelm cellular antioxidant protections and lead to cell death. Further experiments exploring this point will be addressed in the future. 

From our studies, in particular from results obtained with FSK, an important point emerges: stimulation of mitochondrial activity and restoration of an ATP generating mechanism more similar to nonmalignant cells, might be an efficient tool in anticancer strategy. In particular, shifting cellular metabolism towards mitochondrial ATP production might overcome the positive effects on glycolytic pathway of oncogenes like K-ras, Akt and HIF-1 [42]. The idea is not completely new, since other authors have addressed this point by redirection of pyruvate towards oxidation in the mitochondria. In fact, inhibition of pyruvate dehydrogenase kinase (PDK) by DCA, or lactate dehydrogenase (LDH) by RNAi, has been shown to shift metabolism from glycolysis to glucose oxidation and to strongly reduce cancer cell viability and tumor growth [18, 43]. Our results with FSK suggest a similar mechanism in which reactivation of the mitochondrial function associated with glucose depletion and mitochondrial Complex I inhibition strongly affects cancer cell survival. Therefore, strategies involving the manipulation of both glycolytic and mitochondrial pathways might be useful to eradicate cancer cells. Other information in such a direction was derived also by experiments with oligomycin, an inhibitor of mitochondrial ATP synthase. We show that it is able to enhance cancer cell death in low glucose as compared to high glucose (Figure 7). In fact, we observed that in normal glucose conditions it does not induce a strong reduction of cell viability, although it is able to reduce the capability to form colonies. We can suppose that such a long-term effect is due to the inhibition of the reverse action of ATPase [44] that in fact is reflected in the mitochondrial potential decrease not accompanied with loss of ATP. On the other hand, the stronger effect of oligomycin on MDA-MB-231 cells in glucose deprivation is experimental evidence of their forced dependence on OXPHOS in such condition, exploitable by mitochondrial targeting therapies. Moreover, from another point of view, these data could suggest that inhibition of residual mitochondrial activity of cancer cells will further upregulate glycolysis and hence lead to their death by glucose depletion. This finding has been observed in lung carcinoma, in which oligomycin treatment upregulates glycolysis, increasing their dependence on this metabolic pathway [45].

Taken together our results provide a rationale for the use of mitochondrial inhibitors in cancer cells exploiting cancer cell fragility versus glucose depletion. In addition they point to an energetic switch from glycolysis to OXPHOS as an important therapeutic approach, since normal cells appear to be resistant to such combined treatments.

## Supplementary Material

Representative profiles of PI/Annexin V staining for MDA-MB-231 cells treated with rotenone and FSK alone or in combination.
The profiles, together with the quantitative analysis (figure 3C), indicate that FSK treatment of MDA-MB-231 cells enhances the viability loss induced by rotenone alone.

## References

[1] Hanahan D, Weinberg RA (2011). Hallmarks of cancer: the next generation. *Cell*.

[2] DeBerardinis RJ, Lum JJ, Hatzivassiliou G, Thompson CB (2008). The biology of cancer: metabolic reprogramming fuels cell growth and proliferation. *Cell Metabolism*.

[3] Chiaradonna F, Moresco RM, Airoldi C, Gaglio D, Palorini R, Nicotra F (2012). From cancer metabolism to new biomarkers and drug targets. *Biotechnology Advances*.

[4] Jones RG, Thompson CB (2009). Tumor suppressors and cell metabolism: a recipe for cancer growth. *Genes and Development*.

[5] Hsu PP, Sabatini DM (2008). Cancer cell metabolism: warburg and beyond. *Cell*.

[6] Lu J, Sharma LK, Bai Y (2009). Implications of mitochondrial DNA mutations and mitochondrial dysfunction in tumorigenesis. *Cell Research*.

[7] Ralph SJ, Low P, Dong L, Lawen A, Neuzil J (2006). Mitocans: mitochondrial targeted anti-cancer drugs as improved therapies and related patent documents. *Recent Patents on Anti-Cancer Drug Discovery*.

[8] Biasutto L, Dong LF, Zoratti M, Neuzil J (2010). Mitochondrially targeted anti-cancer agents. *Mitochondrion*.

[9] Neuzil J, Dyason JC, Freeman R (2007). Mitocans as anti-cancer agents targeting mitochondria: lessons from studies with vitamin e analogues, inhibitors of complex II. *Journal of Bioenergetics and Biomembranes*.

[10] Pathania D, Millard M, Neamati N (2009). Opportunities in discovery and delivery of anticancer drugs targeting mitochondria and cancer cell metabolism. *Advanced Drug Delivery Reviews*.

[11] Pelicano H, Martin DS, Xu RH, Huang P (2006). Glycolysis inhibition for anticancer treatment. *Oncogene*.

[12] El Mjiyad N, Caro-Maldonado A, Ramírez-Peinado S, Mũoz-Pinedo C (2011). Sugar-free approaches to cancer cell killing. *Oncogene*.

[13] Graham NA, Tahmasian M, Kohli B, Komisopoulou E, Zhu M, Vivanco I (2012). Glucose deprivation activates a metabolic and signaling amplification loop leading to cell death. *Molecular Systems Biology*.

[14] Simons AL, Mattson DM, Dornfeld K, Spitz DR (2009). Glucose deprivation-induced metabolic oxidative stress and cancer therapy. *Journal of Cancer Research and Therapeutics*.

[15] Palorini R, De Rasmo D, Gaviraghi M, Sala Danna L, Signorile A, Cirulli C (2013). Oncogenic K-ras expression is associated with derangement of the cAMP/PKA pathway and forskolin-reversible alterations of mitochondrial dynamics and respiration. *Oncogene*.

[16] Liu H, Hu YP, Savaraj N, Priebe W, Lampidis TJ (2001). Hypersensitization of tumor cells to glycolytic inhibitors. *Biochemistry*.

[17] Fath MA, Diers AR, Aykin-Burns N, Simons AL, Hua L, Spitz DR (2009). Mitochondrial electron transport chain blockers enhance 2-deoxy-D-glucose induced oxidative stress and cell killing in human colon carcinoma cells. *Cancer Biology and Therapy*.

[18] Bonnet S, Archer SL, Allalunis-Turner J (2007). A mitochondria-K^+^ channel axis is suppressed in cancer and its normalization promotes apoptosis and inhibits cancer growth. *Cancer Cell*.

[19] Chiaradonna F, Balestrieri C, Gaglio D, Vanoni M (2008). RAS and PKA pathways in cancer: new insight from transcriptional analysis. *Frontiers in Bioscience*.

[20] Seamon KB, Padgett W, Daly JW (1981). Forskolin: unique diterpene activator of adenylate cyclase in membranes and in intact cells. *Proceedings of the National Academy of Sciences of the United States of America*.

[21] Seon YN, Amoscato AA, Lee YJ (2002). Low glucose-enhanced TRAIL cytotoxicity is mediated through the ceramide-Akt-FLIP pathway. *Oncogene*.

[22] Yun J, Rago C, Cheong I (2009). Glucose deprivation contributes to the development of KRAS pathway mutations in tumor cells. *Science*.

[23] Chiaradonna F, Sacco E, Manzoni R, Giorgio M, Vanoni M, Alberghina L (2006). Ras-dependent carbon metabolism and transformation in mouse fibroblasts. *Oncogene*.

[24] Gaglio D, Metallo CM, Gameiro PA, Hiller K, Danna LS, Balestrieri C (2011). Oncogenic K-Ras decouples glucose and glutamine metabolism to support cancer cell growth. *Molecular Systems Biology*.

[25] Deng YT, Huang HC, Lin JK (2010). Rotenone induces apoptosis in MCF-7 human breast cancer cell-mediated ROS through JNK and p38 signaling. *Molecular Carcinogenesis*.

[26] Bernard G, Bellance N, James D (2007). Mitochondrial bioenergetics and structural network organization. *Journal of Cell Science*.

[27] Betarbet R, Sherer TB, MacKenzie G, Garcia-Osuna M, Panov AV, Greenamyre JT (2000). Chronic systemic pesticide exposure reproduces features of Parkinson’s disease. *Nature Neuroscience*.

[28] Choi WS, Palmiter RD, Xia Z (2011). Loss of mitochondrial complex I activity potentiates dopamine neuron death induced by microtubule dysfunction in a Parkinson’s disease model. *Journal of Cell Biology*.

[29] Li N, Ragheb K, Lawler G (2003). Mitochondrial complex I inhibitor rotenone induces apoptosis through enhancing mitochondrial reactive oxygen species production. *Journal of Biological Chemistry*.

[30] Balestrieri C, Vanoni M, Hautaniemi S, Alberghina L, Chiaradonna F (2011). Integrative transcriptional analysis between human and mouse cancer cells provides a common set of transformation associated genes. *Biotechnology Advances*.

[31] Chiaradonna F, Gaglio D, Vanoni M, Alberghina L (2006). Expression of transforming K-Ras oncogene affects mitochondrial function and morphology in mouse fibroblasts. *Biochimica et Biophysica Acta*.

[32] Okun JG, Lümmen P, Brandt U (1999). Three classes of inhibitors share a common binding domain in mitochondrial complex I (NADH:Ubiquinone oxidoreductase). *Journal of Biological Chemistry*.

[33] Armstrong JS, Hornung B, Lecane P, Jones DP, Knox SJ (2001). Rotenone-induced G2/M cell cycle arrest and apoptosis in a human B lymphoma cell line PW. *Biochemical and Biophysical Research Communications*.

[34] Srivastava P, Panda D (2007). Rotenone inhibits mammalian cell proliferation by inhibiting microtubule assembly through tubulin binding. *FEBS Journal*.

[35] Edinger AL, Thompson CB (2004). Death by design: apoptosis, necrosis and autophagy. *Current Opinion in Cell Biology*.

[36] Zaccagnino P, Corcelli A, Baronio M, Lorusso M (2011). Anandamide inhibits oxidative phosphorylation in isolated liver mitochondria. *FEBS Letters*.

[37] Alberghina L, Gaglio D, Gelfi C, Moresco RM, Mauri G, Bertolazzi P (2012). Cancer cell growth and survival as a system-level property sustained by enhanced glycolysis and mitochondrial metabolic remodeling. *Frontiers in Physiology*.

[38] Alirol E, Martinou JC (2006). Mitochondria and cancer: is there a morphological connection?. *Oncogene*.

[39] Schulz TJ, Thierbach R, Voigt A (2006). Induction of oxidative metabolism by mitochondrial frataxin inhibits cancer growth: otto Warburg revisited. *Journal of Biological Chemistry*.

[40] Hervouet E, Demont J, Pecina P (2005). A new role for the von Hippel-Lindau tumor suppressor protein: stimulation of mitochondrial oxidative phosphorylation complex biogenesis. *Carcinogenesis*.

[41] Pramanik KC, Boreddy SR, Srivastava SK (2011). Role of mitochondrial electron transport chain complexes in capsaicin mediated oxidative stress leading to apoptosis in pancreatic cancer cells. *PLoS ONE*.

[42] Levine AJ, Puzio-Kuter AM (2010). The control of the metabolic switch in cancers by oncogenes and tumor suppressor genes. *Science*.

[43] Le A, Cooper CR, Gouw AM, Dinavahi R, Maitra A, Deck LM (2010). Inhibition of lactate dehydrogenase A induces oxidative stress and inhibits tumor progression. *Proceedings of the National Academy of Sciences of the United States of America*.

[44] Campanella M, Parker N, Tan CH, Hall AM, Duchen MR (2009). IF1: setting the pace of the F1Fo-ATP synthase. *Trends in Biochemical Sciences*.

[45] Wu M, Neilson A, Swift AL (2007). Multiparameter metabolic analysis reveals a close link between attenuated mitochondrial bioenergetic function and enhanced glycolysis dependency in human tumor cells. *American Journal of Physiology*.

