# Supplementary material for: Mitochondrial Complex I Inhibitors and Forced Oxidative Phosphorylation Synergize in Inducing Cancer Cell Death
Source: Int J Cell Biol. 2013 Apr 9;2013:243876. doi: 10.1155/2013/243876 (PMC3638674; doi:10.1155/2013/243876)
Supplement: Supplementary file 1 — Representative profiles of PI/Annexin V staining for MDA-MB-231 cells treated with rotenone and FSK alone or in combination. The profiles, together with the quantitative analysis (figure 3C), indicate that FSK treatment of MDA-MB-231 cells enhances the viability loss induced by rotenone alone. [file 243876.f1.pdf]

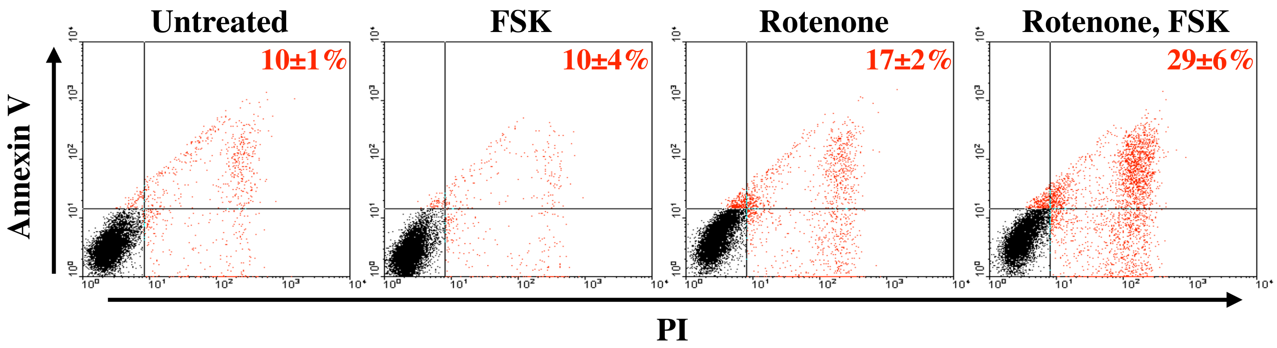

Supplementary figure 1. FSK treatment enhances the viability loss induced by rotenone alone in MDA-MB-231 cells. MDA-MB-231 cells were cultured in 1 mM glucose and treated with 3 nM rotenone, 10 μM forskolin (FSK) or both molecules at 48 hours of culture. Cells were pre-treated for 1 hour with FSK and then rotenone was also added for 4 hours as shown in figure 2C. After treatment cell viability was evaluated staining cells with Propidium Iodide (PI) and Annexin V-FITC; here representative profiles are shown and the percentages (± s.d.) of dead cells (positive for PI and Annexin V) are indicated in red. For quantitative analysis refer also to figure 3. The profiles are indicative of three independent experiments.
